# Ultrasound-Assisted Fermentation by *Lactiplantibacillus plantarum* Promotes Phytochemical Transformation and Antioxidant Activity of *Citrus aurantium* L. Through Metabolic Alterations

**DOI:** 10.3390/foods15132306

**Published:** 2026-06-29

**Authors:** Zhengnan Ren, Ningning Shen, Linxiao Wang, Shun Li, Longquan Xiao, Lin Zhou, Binbin Li, Xinhui Wang

**Affiliations:** College of Food and Biological Engineering, Chengdu University, Chengdu 610106, China; rzhengnan@outlook.com (Z.R.);

**Keywords:** *Citrus aurantium* L, *Lactiplantibacillus plantarum*, ultrasound-assisted fermentation, flavonoid biotransformation, antioxidant activity

## Abstract

*Citrus aurantium* L. is a citrus-derived functional food rich in various phenolic compounds, including flavonoids. However, the bioavailability of its phytochemicals and sensory quality remain limited. This study investigated the effects of ultrasound-assisted fermentation by *Lactiplantibacillus plantarum* on microbial growth, phytochemical transformation, antioxidant activity, and metabolic profiles of *C. aurantium*. Ultrasound treatments were applied at different fermentation stages and power levels, among which treatment at 100 W during the exponential growth phase (ULP4) exhibited the best overall performance. Compared with conventional fermentation by *L. plantarum* (LP), ULP4 significantly promoted microbial growth, accelerated acidification, enhanced carbohydrate utilization, and stimulated β-glucosidase activity compared with conventional fermentation. Consequently, total phenolic content, total flavonoid content, and antioxidant capacities were markedly improved. Untargeted full-MS/MS metabolomic analysis revealed extensive metabolic alterations following ultrasound treatment, with 335 metabolites significantly altered between LP and ULP4. Differential metabolites were mainly associated with flavone and flavonol biosynthesis, secondary metabolite biosynthesis, amino acid metabolism, and lipid metabolism. These metabolic changes were closely associated with improved antioxidant properties and functional quality. Overall, ultrasound treatment during the exponential growth phase effectively enhanced microbial metabolism and phytochemical transformation, offering a promising strategy to improve the functional value of fermented *C. aurantium* products.

## 1. Introduction

*Citrus aurantium* L. is a traditional medicinal and edible citrus widely cultivated for its rich content of flavonoids, phenolic compounds, and essential oils [1,2]. These bioactive constituents have been associated with antioxidant, anti-inflammatory, and cardiovascular protective effects, making *C. aurantium* a promising functional food resource [3,4,5]. Despite its health-promoting properties, the application of *C. aurantium* in food and nutraceutical products is limited by inherent bitterness and low bioavailability of flavonoid glycosides [1,6]. The poor solubility and low extractability of certain bioactive compounds further restrict their functional and sensory utilization, highlighting the need for processing strategies that enhance both nutritional value and palatability.

Fermentation by lactic acid bacteria has been widely applied to plant-based matrices to improve their nutritional, functional, and sensory qualities [7]. In particular, *Lactiplantibacillus plantarum* exhibits the ability to hydrolyze flavonoid glycosides into their aglycone forms via β-glucosidase activity, thereby increasing bioavailability and antioxidant potential [8]. Moreover, microbial metabolism during fermentation can generate desirable volatile compounds and reduce undesirable bitter or astringent notes, contributing to improved flavor profiles [9,10]. In citrus-derived substrates, lactic acid fermentation has been shown to enhance phenolic content, modulate sugar composition, and elevate antioxidant capacity, suggesting that fermentation is an effective tool to valorize underutilized citrus fruits [11].

Ultrasound-assisted fermentation has recently attracted increasing attention as a strategy for stimulating microbial metabolism and enhancing biotransformation efficiency [12,13,14]. Moderate ultrasound treatment can improve mass transfer, increase substrate accessibility, and promote enzyme activities through cavitation effects [15,16]. Previous studies have demonstrated that ultrasound can enhance microbial growth and metabolite production in fermented foods [17,18]. However, most investigations have focused on the presence or absence of ultrasound treatment, whereas the influence of ultrasound application timing during different fermentation phases remains poorly understood [19]. Given that microbial physiology and metabolic activity vary substantially throughout fermentation, ultrasound intervention at different growth stages may result in distinct biotransformation efficiencies and metabolite profiles. A better understanding of phase-dependent ultrasound responses could therefore provide an effective strategy for optimizing fermentation efficiency and product quality in industrial production.

Therefore, this study investigated the effects of ultrasound treatment applied at different fermentation stages on *L. plantarum*-mediated fermentation of *C. aurantium*. Particular attention was given to microbial growth, β-glucosidase activity, phytochemical transformation, antioxidant capacity, and metabolic alterations. By integrating biochemical analyses with untargeted metabolomics, this work aimed to elucidate the influence of phase-dependent ultrasound intervention on microbial metabolism and phytochemical transformation, while providing practical insights for improving the efficiency and functional quality of fermented citrus products.

## 2. Materials and Methods

### 2.1. Materials and Chemicals

*Citrus aurantium* L. fruits were purchased from Sichuan Jinfang Biomedicine Technology Co., Ltd. (Guang’an, China). Fresh fruits were washed, peeled, and homogenized to prepare the substrate. *L. plantarum* strain ATCC 14917 was obtained from ATCC (Manassas, VA, USA). All chemicals and standards were purchased from Merck (Shanghai, China).

### 2.2. Preparation of Fruit Substrate

The homogenized *C. aurantium* pulp was mixed with distilled water at a solid-to-liquid ratio of 1:3 *w*/*v*, and the mixture was pasteurized at 85 °C for 15 min to inactivate endogenous enzymes and native microorganisms. After cooling to room temperature, the pH was adjusted to pH 6.5. The substrate was then divided into experimental batches for subsequent ultrasound and fermentation treatments.

### 2.3. Ultrasound-Assisted Fermentation

*L. plantarum* was first activated in MRS broth at 37 °C for 18 h. The culture was then centrifuged, washed twice with sterile saline, and resuspended for inoculation. The prepared *C. aurantium* substrate was inoculated with 3% (*v*/*v*) *L. plantarum* suspension and mixed thoroughly. Ultrasound treatment was performed using a probe-type ultrasonic processor (SCIENTZ-IID, Ningbo Scientz Biotechnology Co., Ltd., Ningbo, China) equipped with a 6 mm titanium probe and operated at a frequency of 20 kHz. Ultrasound treatments were applied at power levels of 60 W or 100 W in an intermittent mode (5 s on/5 s off) for a total treatment time of 5 min. The sample temperature was maintained using an ice bath to minimize thermal effects. According to the ultrasound treatment conditions, the samples were divided into six groups: ULP1 (60 W, ultrasound applied at 0 h), ULP2 (100 W, ultrasound applied at 0 h), ULP3 (60 W, ultrasound applied at 8 h), ULP4 (100 W, ultrasound applied at 8 h), ULP5 (60 W, ultrasound applied at 18 h), and ULP6 (100 W, ultrasound applied at 18 h). A conventional fermentation by *L*. *plantarum* was used as the control (LP). All samples were subsequently incubated at 37 °C for fermentation. Fermentation samples were collected at 0, 12, 24, and 36 h to monitor pH, viable cell counts, and sugar consumption. Based on the fermentation performance, β-glucosidase activity, total phenolic content, total flavonoid content, antioxidant activity, and metabolomic profiles were determined in samples collected after 36 h of fermentation.

### 2.4. Determination of pH and Viable Cell Counts

The pH values of fermented *Citrus aurantium* L. samples were measured using a calibrated digital pH meter (PHS-2F, INESA Scientific Instrument, Shanghai, China) at room temperature [20]. Measurements were performed at 0, 8, and 18 h of fermentation. For viable cell counts, 1 mL of each sample was serially diluted with sterile saline solution (0.85%, *w*/*v*), and appropriate dilutions were spread onto MRS agar plates [20]. The plates were incubated at 37 °C for 24 h, and then colony counts were conducted.

### 2.5. Determination of Total Sugar and Reducing Sugar

Total sugar and reducing sugar contents were determined using commercial assay kits (BC2710, BC0230; Solarbio, Beijing, China) according to the manufacturer’s instructions. Briefly, appropriately diluted samples were reacted with the corresponding reagents provided in the kits, and absorbance was measured at OD_540_ using a microplate reader (Spectramax i3x, Molecular Devices, CA, USA).

### 2.6. Determination of Total Phenolic Content, Total Flavonoid Content, and Total Antioxidant Capacity

Total phenolic content (TPC) and total flavonoid content (TFC) were determined using commercial assay kits (BC1340, BC1330; Solarbio) following the manufacturer’s protocols. Absorbance values were recorded using a microplate reader (Spectramax i3x).

### 2.7. Determination of β-Glucosidase Activity

β-glucosidase activity was measured using a commercial assay kit (BC2560, Solarbio) according to the manufacturer’s instructions. Absorbance was measured at OD_400_ using a microplate reader.

### 2.8. Determination of Antioxidant Activity

Antioxidant activity was evaluated using DPPH and ABTS radical scavenging assay kits (BC4750, BC4770; Solarbio) according to the manufacturer’s instructions. Absorbance values were measured at OD_515_ for DPPH and OD_405_ for ABTS using a microplate reader.

### 2.9. LC-MS and GC-MS Analysis

Untargeted metabolomic and volatile compound analyses were performed by Majorbio Bio-Pharm Technology Co., Ltd. (Shanghai, China). LC-MS/MS analysis was conducted using a UHPLC-Q Exactive HF-X system (Thermo Fisher Scientific, Vacaville, CA, USA) equipped with an HSS T3 column (100 mm × 2.1 mm, 1.8 μm; Waters, Milford, MA, USA). The mobile phases consisted of solvent A [0.1% formic acid in water/acetonitrile (95:5, *v*/*v*)] and solvent B [0.1% formic acid in acetonitrile/isopropanol/water (47.5:47.5:5, *v*/*v*/*v*)]. The flow rate was 0.40 mL/min and the column temperature was maintained at 40 °C. Mass spectrometric data were acquired in both positive and negative electrospray ionization (ESI) modes with a scan range of *m*/*z* 70–1050.

GC-MS analysis was performed using a TRACE 1610 GC-Orbitrap Exploris system (Thermo Fisher Scientific) equipped with a TG-5SILMS capillary column (30 m × 0.25 mm × 0.25 μm). Helium was used as the carrier gas at a flow rate of 1.0 mL/min. The oven temperature was initially set at 80 °C and increased to 310 °C at a rate of 20 °C/min, followed by holding for 8 min. Electron impact ionization was operated at 70 eV with a mass scan range of *m*/*z* 35–500.

Raw LC-MS data were processed using Progenesis QI software (Waters Corporation, Milford, MA, USA) for peak detection, extraction, alignment, and integration. Metabolite annotation was performed by matching MS and MS/MS spectra against the HMDB, Metlin, and Majorbio self-built databases. After merging the LC-MS and GC-MS datasets and removing duplicate metabolites, the combined data matrix was uploaded to the Majorbio Cloud Platform https://cloud.majorbio.com for subsequent analyses. Features with more than 20% missing values in each group were removed, and missing values were imputed using minimum value replacement. The mass spectral peak intensities were normalized using the sum normalization method, followed by exclusion of variables with a relative standard deviation (RSD) > 30% in QC samples. The data were subsequently log10-transformed to generate the final normalized data matrix for downstream analyses.

### 2.10. Statistical Analysis

All experiments were performed in triplicate, and the results were expressed as mean ± standard deviation (SD). Statistical analyses and graph preparation were conducted using GraphPad Prism (10.1.2, San Diego, CA, USA). Significant differences among groups were evaluated using one-way analysis of variance (ANOVA) followed by Tukey’s multiple comparison test, with *p* < 0.05 considered statistically significant. Multivariate statistical analyses of metabolomic and volatile compound data were performed using the Majorbio Cloud Platform. Correlation analysis and heatmap visualization were also conducted to evaluate relationships among physicochemical properties, antioxidant activity, and metabolite profiles.

## 3. Results and Discussion

### 3.1. Effects of Ultrasound-Assisted Fermentation on the Growth and Acidification of Lactiplantibacillus plantarum

**Figure 1:**
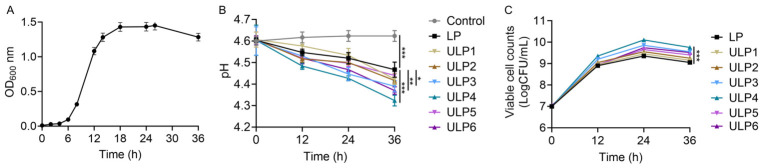
Effects of ultrasound-assisted fermentation on the growth and acidification of *L. plantarum*. (**A**) Growth curve of *L. plantarum*. (**B**) Changes in pH during fermentation. (**C**) Changes in viable cell counts during fermentation. LP, conventional fermentation without ultrasound; ULP1–ULP6, ultrasound-assisted fermentation treatments performed at different fermentation stages and power levels. Data are presented as mean ± SD (*n* = 3). Statistical significance in (**B**,**C**) among treatments was determined by two-way ANOVA followed by Tukey’s multiple comparison test (* *p* < 0.05, ** *p* < 0.01, *** *p* < 0.001).

*L. plantarum* exhibited a typical growth pattern during fermentation, consisting of a lag phase (0–6 h), an exponential phase (6–14 h), and a stationary phase (18–27 h), followed by a slight decline at 36 h (Figure 1A). Based on the growth kinetics, ultrasound treatments were applied at 0 h (lag phase), 8 h (early exponential phase), and 18 h (stationary phase) to investigate the influence of ultrasound intervention at different physiological stages of bacterial growth. The pH of all fermentation groups gradually decreased throughout fermentation, indicating continuous acid production by *L. plantarum* (Figure 1B). Compared with the conventional fermentation group (LP), ultrasound-assisted fermentation accelerated acidification, particularly in the 100 W treatment groups. After 36 h of fermentation, ULP4 exhibited the lowest pH value (4.32), followed by ULP6 (4.37), whereas LP remained at 4.47. The lower pH values observed in ultrasound-treated groups suggest enhanced acidification during fermentation, which may be associated with changes in microbial activity and metabolite production. Similar effects have been reported in previous studies, where ultrasound treatment promoted microbial fermentation and accelerated acid accumulation [21]. Furthermore, viable cell counts increased rapidly during the first 24 h and subsequently decreased slightly at 36 h (Figure 1C). All ultrasound-treated groups exhibited higher viable counts than LP throughout fermentation. Among them, ULP4 achieved the highest cell density, indicating that ultrasound treatment applied during the early exponential phase was more favorable for bacterial growth. In contrast, ultrasound treatments applied at the beginning of fermentation (ULP1 and ULP2) resulted in relatively smaller improvements. At 36 h, viable cell counts remained significantly higher in ULP4 and ULP6 than in LP. These results suggest that appropriately timed ultrasound treatment may promote microbial growth and fermentation performance, with the greatest effect observed when ultrasound was applied during the exponential growth phase.

### 3.2. Effects of Ultrasound-Assisted Fermentation on Carbohydrate Utilization

**Figure 2:**
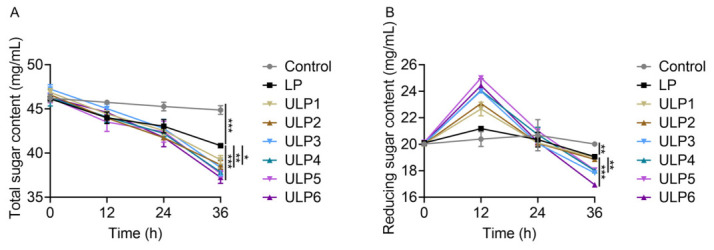
Effects of ultrasound-assisted fermentation on carbohydrate utilization during *C. aurantium* fermentation. (**A**) Changes in total sugar content during fermentation. (**B**) Changes in reducing sugar content during fermentation. LP, conventional fermentation without ultrasound; ULP1–ULP6, ultrasound-assisted fermentation treatments performed at different fermentation stages and power levels. Data are presented as mean ± SD (*n* = 3). Statistical significance in (**A**,**B**) among treatments was determined by two-way ANOVA followed by Tukey’s multiple comparison test (* *p* < 0.05, ** *p* < 0.01, *** *p* < 0.001).

The total sugar content decreased progressively during fermentation in all groups, indicating continuous carbohydrate consumption by *L. plantarum* (Figure 2A). Compared with LP, all ultrasound-treated groups exhibited lower residual total sugar contents after 36 h [21]. Among them, ULP6 showed the lowest total sugar content (37.24 mg/mL), followed by ULP5 (37.67 mg/mL) and ULP4 (37.84 mg/mL), whereas LP retained 40.83 mg/mL. Reducing sugar content initially increased and subsequently decreased during fermentation (Figure 2B). The increase observed at 12 h may be attributed to the hydrolysis of complex carbohydrates into fermentable sugars, while the subsequent decline reflected microbial consumption [22]. At 36 h, all ultrasound-treated groups exhibited lower reducing sugar contents than LP, with ULP6 showing the lowest value (16.93 mg/mL). The lower residual total and reducing sugar contents observed in ultrasound-treated groups suggest that ultrasound treatment influenced fermentation dynamics and sugar conversion during the fermentation process [23]. Notably, ULP4 achieved sugar depletion comparable to ULP6 while exhibiting superior cell growth and acidification performance, indicating that ultrasound application during the exponential phase was associated with improved fermentation performance.

### 3.3. Effects of Ultrasound-Assisted Fermentation on β-Glucosidase Activity

**Figure 3:**
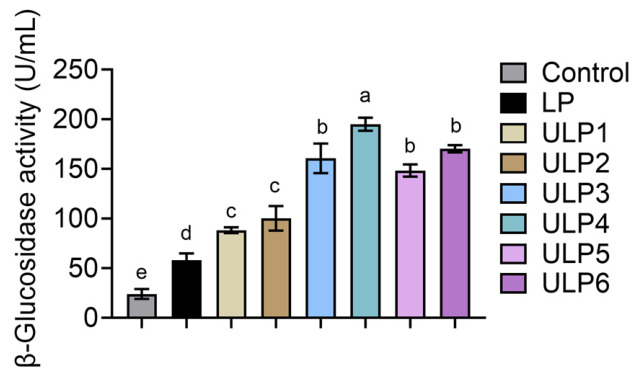
Effects of ultrasound-assisted fermentation on β-glucosidase activity during *C. aurantium* fermentation. LP, conventional fermentation without ultrasound; ULP1–ULP6, ultrasound-assisted fermentation treatments performed at different fermentation stages and power levels. Data are presented as mean ± SD (*n* = 3). Different lowercase letters above the bars indicate significant differences among treatments according to one-way ANOVA followed by Tukey’s multiple comparison test (*p* < 0.05).

To further investigate the effect of ultrasound on enzymatic biotransformation, β-glucosidase activity was determined, as this enzyme plays a crucial role in the hydrolysis of flavonoid glycosides and the formation of bioactive aglycones [24]. As shown in Figure 3, fermentation significantly enhanced β-glucosidase activity compared with the control, while ultrasound-assisted fermentation was associated with a further enhancement of enzyme activity. All ultrasound-treated groups exhibited significantly higher β-glucosidase activities than the LP group, suggesting that ultrasound treatment influenced enzymatic transformation during fermentation [25]. The enhancement of β-glucosidase activity was strongly influenced by the timing of ultrasound application. Among all treatments, ULP4 exhibited the highest enzyme activity and differed significantly from the other groups. In contrast, ULP3, ULP5, and ULP6 showed intermediate activities, whereas ULP1 and ULP2 displayed relatively lower enzyme activities. These results indicate that ultrasound treatment applied during the exponential growth phase was more effective in enhancing β-glucosidase activity than treatments applied during the lag or stationary phases. Previous studies have reported that moderate ultrasound treatment can enhance enzyme activities and fermentation performance in microbial fermentation systems [26]. In addition, β-glucosidase produced by *L. plantarum* plays an important role in the conversion of flavonoid glycosides into bioactive aglycones. Therefore, the elevated β-glucosidase activity observed in ULP4 may have facilitated flavonoid biotransformation during fermentation, which is consistent with the increased phenolic content and antioxidant activity observed in this study.

### 3.4. Effects of Ultrasound-Assisted Fermentation on Total Phenolic and Flavonoid Contents

**Figure 4:**
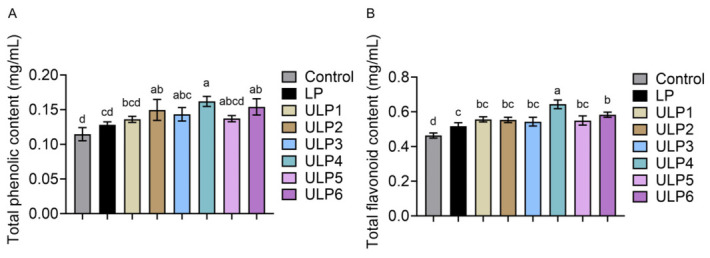
Effects of ultrasound-assisted fermentation on phytochemical accumulation during *C. aurantium* fermentation. (**A**) Total phenolic content. (**B**) Total flavonoid content. LP, conventional fermentation without ultrasound; ULP1–ULP6, ultrasound-assisted fermentation treatments performed at different fermentation stages and power levels. Data are presented as mean ± SD (*n* = 3). Different lowercase letters above the bars indicate significant differences among treatments according to one-way ANOVA followed by Tukey’s multiple comparison test (*p* < 0.05).

The enhanced β-glucosidase activity observed in ultrasound-treated groups may facilitate the release and biotransformation of phenolic compounds during fermentation [8]. Therefore, the total phenolic content (TPC) and total flavonoid content (TFC) were further evaluated to assess the effects of ultrasound-assisted fermentation on phytochemical accumulation. The fermentation increased TPC compared with the control, and ultrasound treatment further promoted phenolic accumulation (Figure 4A). Among all treatments, ULP4 exhibited the highest TPC, followed by ULP6 and ULP2. The increase in TPC may be attributed to ultrasound-induced cavitation, which facilitates the disruption of plant cell structures and promotes the release of bound phenolic compounds [27,28]. Similar observations have been reported in previous studies on ultrasound-assisted processing of plant materials. Ultrasound treatment has been shown to enhance the extraction, release, and accumulation of phenolic compounds by improving cell disruption and mass transfer efficiency. For example, ultrasound-assisted extraction significantly increased the recovery of phenolic compounds and antioxidant activity in Citrus aurantium and other plant-based functional foods [29,30]. These findings are consistent with the enhanced phenolic accumulation observed in the present study. Moreover, a similar but more pronounced trend was observed for TFC (Figure 4B). ULP4 exhibited the highest TFC and was significantly higher than all other treatments, while ULP6 showed the second-highest value. Notably, the variation in TFC closely paralleled the changes in β-glucosidase activity, suggesting that ultrasound-assisted fermentation enhanced flavonoid biotransformation through enzymatic hydrolysis of flavonoid glycosides. Since flavonoids represent a major class of bioactive compounds in *C. aurantium*, the elevated TFC observed in ULP4 indicates that ultrasound application during the exponential growth phase most effectively promoted the release and conversion of flavonoid compounds [2]. These results demonstrate that the timing of ultrasound treatment plays a critical role in phytochemical transformation during fermentation. The superior accumulation of phenolic and flavonoid compounds in ULP4 provides a biochemical basis for the subsequent enhancement of antioxidant activity and functional properties.

### 3.5. Effects of Ultrasound-Assisted Fermentation on Antioxidant Activity

**Figure 5:**
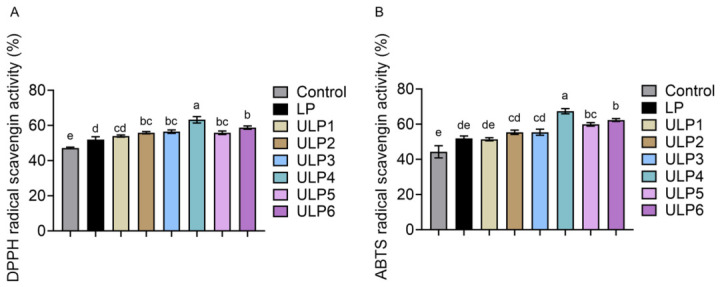
Effects of ultrasound-assisted fermentation on the antioxidant activity of *C. aurantium*. (**A**) DPPH radical scavenging activity. (**B**) ABTS radical scavenging activity. LP, conventional fermentation without ultrasound; ULP1–ULP6, ultrasound-assisted fermentation treatments performed at different fermentation stages and power levels. Data are presented as mean ± SD (*n* = 3). Different lowercase letters above the bars indicate significant differences among treatments according to one-way ANOVA followed by Tukey’s multiple comparison test (*p* < 0.05).

The accumulation of phenolic and flavonoid compounds of plants during fermentation is closely associated with enhanced antioxidant capacity [31]. Therefore, DPPH and ABTS radical scavenging activities were determined to evaluate the effects of ultrasound-assisted fermentation on the antioxidant properties of *C. aurantium* [32]. Fermentation markedly improved both DPPH and ABTS radical scavenging activities relative to the control (Figure 5A,B), indicating that microbial metabolism promoted the formation and release of antioxidant compounds. Ultrasound treatment further enhanced antioxidant activity, although the magnitude of the improvement depended on the timing and power of ultrasound application. Among all treatments, ULP4 exhibited the strongest radical scavenging capacities. The variation in antioxidant activity closely paralleled the changes observed in β-glucosidase activity and flavonoid accumulation. Enhanced β-glucosidase activity may accelerate the conversion of flavonoid glycosides into corresponding aglycones, which generally possess greater antioxidant potential [33]. In addition, ultrasound-induced cavitation can facilitate cell wall disruption and improve the release of bound phenolic compounds from the plant matrix, thereby increasing the availability of antioxidant constituents [34,35]. These effects collectively contributed to the superior antioxidant performance of ultrasound-treated samples. Similar improvements in antioxidant activity have been reported in previous ultrasound-assisted fermentation and processing studies. Ultrasound treatment has been shown to enhance the release and transformation of phenolic compounds and flavonoids, resulting in increased radical scavenging capacity and antioxidant potential. The consistency between these reports and the present findings further supports the effectiveness of ultrasound-assisted fermentation in improving the functional quality of plant-based products [20,36]. Notably, ultrasound application at 100 W during the exponential growth phase (ULP4) resulted in the highest antioxidant activity, suggesting that this treatment most effectively promoted phytochemical transformation during fermentation.

### 3.6. Untargeted Metabolomic Profiling of LP and ULP4

**Figure 6:**
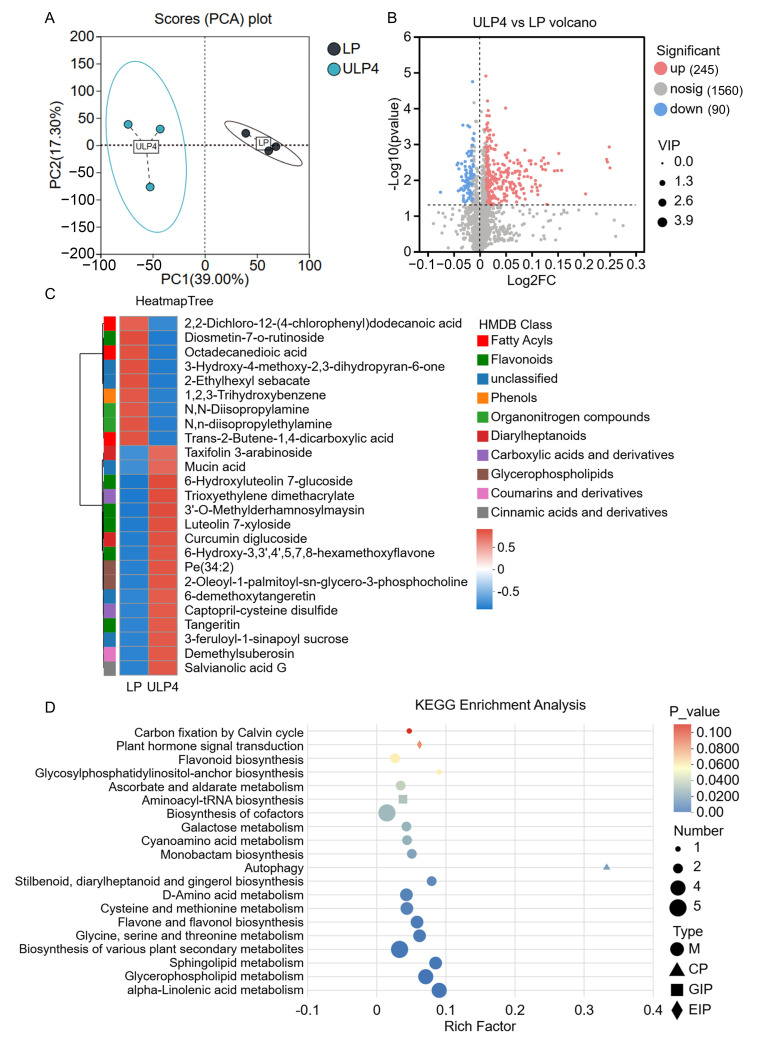
Untargeted metabolomic analysis of LP and ULP4 samples. (**A**) Principal component analysis (PCA) score plot showing the metabolic separation between LP and ULP4. (**B**) Volcano plot of differential metabolites between LP and ULP4. Red and blue dots represent significantly up- and down-regulated metabolites, respectively, while gray dots indicate non-significant metabolites. (**C**) Hierarchical clustering heatmap of representative differential metabolites annotated according to HMDB classification. Red and blue colors indicate relatively high and low metabolite abundance, respectively. (**D**) KEGG pathway enrichment analysis of significantly altered metabolites. Bubble size represents the number of enriched metabolites, and bubble color indicates the enrichment significance level (*p* value). Three biological replicates were analyzed for each group (*n* = 3).

To obtain a comprehensive understanding of the metabolic alterations induced by ultrasound-assisted fermentation, untargeted full-MS/MS metabolomic analysis based on combined LC-MS and GC-MS platforms was performed on LP and ULP4 samples. Principal component analysis (PCA) revealed a clear separation between LP and ULP4, indicating that ultrasound treatment at 100 W during the exponential growth phase substantially altered the metabolic profile of fermented *C. aurantium* (Figure 6A). PC1 and PC2 explained 39.0% and 17.3% of the total variance, respectively, together accounting for 56.3% of the total metabolic variation. Meanwhile, the tight clustering of biological replicates demonstrated the good reproducibility and reliability of the analytical method. Consistent with the PCA results, volcano plot analysis identified 335 significantly altered metabolites between LP and ULP4, including 245 upregulated and 90 downregulated metabolites in ULP4 (Figure 6B and Table S1). These findings suggest that ultrasound-assisted fermentation induced extensive metabolic alterations and significantly influenced metabolite accumulation patterns. Given the marked metabolic differences observed between LP and ULP4, differential metabolites and their associated metabolic pathways were further analyzed to elucidate the phytochemical transformation mechanisms underlying the improved functional properties of ultrasound-assisted fermentation.

### 3.7. Ultrasound Promotes Phytochemical Transformation and Secondary Metabolite Biosynthesis

To further elucidate the metabolic basis underlying the improved functional properties of ULP4, representative differential metabolites and enriched metabolic pathways were analyzed. Hierarchical clustering analysis revealed distinct metabolite accumulation patterns between LP and ULP4 (Figure 6C). Differential metabolites were mainly represented by flavonoids, phenolic compounds, and lipid-related metabolites. Among them, several flavonoid and phenolic compounds, including tangeretin, 6-demethoxytangeretin, salvianolic acid G, curcumin diglucoside, and luteolin derivatives, were enriched in ULP4, whereas diosmetin-7-*O*-rutinoside and naringin exhibited lower abundances. Notably, naringin and diosmetin-7-O-rutinoside are representative flavonoid glycosides commonly found in *Citrus* species. Their depletion of flavonoid glycosides, together with the elevated β-glucosidase activity observed in ULP4 (Figure 3), suggests enhanced hydrolysis and biotransformation of glycosylated flavonoids during ultrasound-assisted fermentation. In contrast, the accumulation of polymethoxylated flavonoids such as tangeretin and 6-demethoxytangeretin, as well as phenolic compounds including salvianolic acid G, may contribute to the enhanced antioxidant activity observed in ULP4 because these compounds have been reported to possess strong antioxidant and bioactive properties. The enrichment of flavonoid-derived metabolites was consistent with the increased TFC and antioxidant activities observed in ULP4 (Figure 4 and Figure 5). Previous studies have demonstrated that the conversion of flavonoid glycosides into lower-molecular-weight derivatives can improve their bioavailability and antioxidant capacity [37]. Therefore, the enhanced β-glucosidase activity induced by ultrasound treatment likely accelerated flavonoid transformation, contributing to the superior functional properties of ULP4.

KEGG pathway enrichment analysis further demonstrated that the differential metabolites were primarily involved in flavone and flavonol biosynthesis, biosynthesis of various plant secondary metabolites, glycine, serine, and threonine metabolism, glycerophospholipid metabolism, and α-linolenic acid metabolism (Figure 6D). These pathways exhibited relatively high enrichment levels, as reflected by their rich factor values and enrichment significance in the KEGG analysis. Among these pathways, flavonoid-related and secondary metabolite biosynthetic pathways were particularly relevant to the enhanced phytochemical accumulation observed in ULP4. In addition, the enrichment of amino acid metabolism may reflect altered microbial metabolic activity during fermentation, while changes in lipid-related pathways may be associated with membrane adaptation and the formation of aroma-related compounds [38]. Collectively, these findings indicate that ultrasound treatment during the exponential growth phase induced substantial metabolic alterations and promoted phytochemical transformation, thereby enhancing the functional quality of fermented *C. aurantium*.

## 4. Conclusions

This study demonstrated that the effectiveness of ultrasound-assisted fermentation of *C. aurantium* was highly dependent on the timing of ultrasound application. Among the tested treatments, ultrasound application at 100 W during the exponential growth phase of *L. plantarum* (ULP4) was identified as the optimal strategy for enhancing fermentation performance and phytochemical transformation. Ultrasound stimulation promoted the metabolic activity of *L. plantarum*, resulting in enhanced carbohydrate utilization and increased β-glucosidase activity, which facilitated the conversion of flavonoid glycosides into more bioactive metabolites. Untargeted full-MS/MS metabolomic analysis revealed substantial metabolic alterations in response to ultrasound-assisted fermentation, particularly in pathways associated with flavonoid biosynthesis, secondary metabolite biosynthesis, and amino acid metabolism. These metabolic alterations contributed to the accumulation of antioxidant-related compounds and were closely associated with the enhanced functional properties of fermented *C. aurantium*. The depletion of flavonoid glycosides together with the enrichment of bioactive flavonoid and phenolic metabolites further confirmed that ultrasound-assisted fermentation promoted phytochemical biotransformation through modulation of microbial metabolism. Overall, the combination of ultrasound treatment and *L. plantarum* fermentation provides an effective approach for directing metabolite conversion and improving the functional quality of *C. aurantium*. This work offers new insights into the synergistic interaction between ultrasound stimulation and microbial fermentation and provides a theoretical basis for the development of value-added fermented citrus products.

## Data Availability

The datasets supporting the conclusions of this study are provided within the article. Any further inquiries regarding materials or data availability may be directed to the authors.

## Supplementary Materials

The following supporting information can be downloaded at: https://www.mdpi.com/article/10.3390/foods15132306/s1, Table S1: Differential metabolites between LP and ULP4.

## Abbreviations

The following abbreviations are used in this manuscript:

*C*. *aurantium*	*Citrus aurantium* L.
*L*. *plantarum*	*Lactiplantibacillus plantarum*
TPC	Total phenolic content
TFC	Total flavonoid content
DPPH	2,2-Diphenyl-1-picrylhydrazyl
ABTS	2,2′-azino-bis (3-ethylbenzothiazoline-6-sulfonic acid)
LC-MS	Liquid Chromatograph/Mass Spectrometer
GC-MS	Gas chromatography–mass spectrometry
RSD	relative standard deviation
SD	standard deviation
ANOVA	analysis of variance
LP	*Lactiplantibacillus plantarum* fermentation without ultrasound treatment
ULP1	*L. plantarum* fermentation with ultrasound treatment at 60 W applied at 0 h
ULP2	*L. plantarum* fermentation with ultrasound treatment at 100 W applied at 0 h
ULP3	*L. plantarum* fermentation with ultrasound treatment at 60 W applied at 8 h
ULP4	*L. plantarum* fermentation with ultrasound treatment at 100 W applied at 8 h
ULP5	*L. plantarum* fermentation with ultrasound treatment at 60 W applied at 18 h
ULP6	*L. plantarum* fermentation with ultrasound treatment at 100 W applied at 18 h
